# Multisite Qualitative Study of Primary Care Physicians’ and Midlevel Providers’ Self-Reported Practices and Perceptions About Maintaining Cognitive Health

**DOI:** 10.5888/pcd9.120050

**Published:** 2012-11-21

**Authors:** Angela K. Hochhalter, Lucinda L. Bryant, Rebecca Hunter, Rui Liu, Daniela B. Friedman, Anna E. Price, Joseph Sharkey, Swarna Reddy, Anthony J. Caprio, Sindy McCrystle

**Affiliations:** Author Affiliations: Lucinda L. Bryant, Colorado School of Public Health, University of Colorado Anschutz Medical Campus, Aurora, Colorado; Rebecca Hunter, Anthony J. Caprio, University of North Carolina at Chapel Hill, Chapel Hill, North Carolina; Rui Liu, National Institute of Environmental Health Sciences, Research Triangle Park, North Carolina; Daniela B. Friedman, University of South Carolina, Columbia, South Carolina; Anna E. Price, Sacred Heart University, Fairfield, Connecticut; Joseph Sharkey, School of Rural Public Health Texas A&M University, College Station, Texas; Swarna Reddy, North Carolina Division of Aging and Adult Services, Raleigh, North Carolina; Sindy McCrystle, Carolinas Medical Center, Charlotte, North Carolina.

## Abstract

**Introduction:**

To facilitate national efforts to maintain cognitive health through public health practice, the Healthy Brain Initiative recommended examining diverse groups to identify stakeholder perspectives on cognitive health. In response, the Healthy Aging Research Network (HAN), funded by the Centers for Disease Control and Prevention (CDC), coordinated projects to document the perspectives of older adults, caregivers of people with dementia, and primary care providers (PCPs) on maintaining cognitive health. Our objective was to describe PCPs’ perceptions and practices regarding cognitive health.

**Methods:**

HAN researchers conducted 10 focus groups and 3 interviews with physicians (N = 28) and advanced practice providers (N = 21) in Colorado, Texas, and North Carolina from June 2007 to November 2008. Data were transcribed and coded axially.

**Results:**

PCPs reported addressing cognitive health with patients only indirectly in the context of physical health or in response to observed functional changes and patient or family requests. Some providers felt evidence on the efficacy of preventive strategies for cognitive health was insufficient, but many reported suggesting activities such as games and social interaction when queried by patients. PCPs identified barriers to talking with patients about cognitive health such as lack of time and patient reactions to recommendations.

**Conclusion:**

Communicating new evidence on cognitive health and engaging older adults in making lasting lifestyle changes recommended by PCPs and others may be practical ways in which public health practitioners can partner with PCPs to address cognitive health in health care settings.

## Introduction

In 2007 as part of the Healthy Brain Initiative, the Centers for Disease Control and Prevention (CDC) and the Alzheimer’s Association released a “road map” for cognitive health, which includes recommendations for incorporating cognitive health into national public health practice (1). The CDC-funded Healthy Aging Research Network (HAN) initiated projects addressing the road map recommendation to describe how diverse groups perceive cognitive health and the associations they make between cognitive health and lifestyle.

In the first HAN project, focus groups with older adults and caregivers of people with cognitive impairment documented perceptions of cognitive health in relation to aging well. Across racial and ethnic groups, older adults and their caregivers defined successful cognitive aging as absence of cognitive impairment, staying “sharp” and “clear minded,” having a good memory, and staying involved in stimulating activities like games (2,3). They believed health behaviors and physical health are tied to cognitive health (4). In the second project, Day and colleagues (5) developed and deployed a 5-item module on cognitive impairment and dementia in the 2008 Porter Novelli DocStyles survey of primary care physicians specializing in family or internal medicine who had been practicing for at least 3 years. Approximately 40% of physicians reported discussing cognition with patients not displaying cognitive impairment either “often” or “very often.” Physicians most commonly reported advising patients to engage in physical activity to prevent cognitive decline and dementia, regardless of perceived strength of evidence for strategies to prevent cognitive impairment. Lack of time and lack of reimbursement were the most commonly cited barriers to discussing prevention of cognitive decline or dementia with primary care patients.

We conducted a qualitative study to build on the first project conducted with older adults and caregivers to provide primary care providers’ (PCPs’) perspectives on similar issues and to add depth and context for findings from PCPs reported by Day et al (5). The study addressed 4 questions: 1) Do PCPs talk about cognitive health with patients who have physical complaints and also are at risk for cognitive impairment?; 2) What recommendations do PCPs believe they can make to help patients maintain cognitive health, and do they make these recommendations in their practices?; 3) What prompts PCPs to initiate discussions about cognitive health?; and 4) What are the barriers to PCPs discussing cognitive health with patients? The objective of this study was to describe PCPs’ perceptions and practices related to cognitive health.

## Methods

Participants were a purposive sample of family practice and internal medicine physicians, nurse practitioners, and physician assistants in Colorado, Texas, and North Carolina recruited through HAN investigators at the Colorado School of Public Health, Texas A&M Health Science Center, and University of North Carolina at Chapel Hill in partnership with University of South Carolina. The sampling frame included providers actively engaged in community-based patient care in urban and rural settings and who were diverse in terms of age, race/ethnicity, and years of practice.

Area Health Education Center (AHEC) partners assisted with recruitment at Colorado and North Carolina sites. The partner AHEC in Colorado sent e-mail invitations to its local provider contact list (approximately 40 people). The partner AHEC in North Carolina e-mailed providers who were registered for professional conferences where focus groups were scheduled to occur. In Texas, invitations to participate were distributed by e-mail or personally from an investigator affiliated with a health care system and by physician champions. Invited physicians in Texas practiced at 2 clinics (estimated 50 physicians invited) or were affiliated with a local physician organization (number invited is unknown). Physician assistants and nurse practitioners (advanced practice providers [APPs]) in Texas were invited through a brief group announcement at a professional conference (approximately 100 APPs invited). Potential participants received invitations to take part in focus groups about brain health as part of the Healthy Brain Study (6,7). All interested providers were either included in focus groups or interviewed.

Focus groups and in-person interviews were conducted from June 2007 to November 2008 in clinics and at professional conferences; 5 focus groups and 3 in-person interviews were conducted with 28 physicians, and 5 focus groups were conducted with 21 APPs. Interviews were conducted with 1 facilitator, 1 note taker, and 1 interviewee. An interviewer (A.K.H., J.S., L.L.B., R.H., S.M.) and a note taker (A.K.H., J.S., L.L.B., S.R.) from the study team attended all focus groups and interviews. Interviews occurred when only 1 participant either came to a scheduled focus group session or indicated interest in the study. Interviews differed from focus groups only in the number of participants and were arranged at the convenience of participants. 

Experienced moderators facilitated focus groups and interviews in English by using a discussion guide developed by the multisite team. Sessions lasted 40 to 60 minutes and were audio recorded. Moderators used probes to obtain and clarify participant responses (8). Research protocols were approved by appropriate institutional review boards.

Although invitations to participate indicated that the project was about cognitive health, the session began with a written case study about a common patient care situation that might suggest but did not specify a higher-than-average long-term risk for cognitive impairment. It was designed to elicit responses exploring the degree to which cognitive health is considered during the course of addressing chronic illnesses such as diabetes:

This case scenario involves a health maintenance visit, presumably at least 30 minutes long.

Mr Henry Y. is a 55-year-old man seen today for a health maintenance visit. Mr Y. is obese and has poorly controlled type 2 diabetes. He has a history of reasonable medication adherence but poor adherence to diet and exercise recommendations. You also care for his mother who is aged 76 with moderate cognitive impairment and severe peripheral neuropathy.

In the context of this visit, what advice (recommendations) will you give him to help maintain his health?

Participants also discussed 6 additional questions about perceptions of aging well, messages they give to patients about cognitive health and prevention of cognitive impairment, perceived value of and barriers to such advice, perceptions of stigma of dementia, and preferred sources of continuing education about cognitive health. This study only examined responses to the case study and the following 2 questions:

Is there anything that physicians could tell their patients to do to help keep their brains healthy, to avoid cognitive decline and/or dementia?

We recognize that physicians face constraints in the time they have to talk with patients. With these constraints in mind: Describe things you actually tell your patients to do to keep their brains healthy.

Coding and data management have been described in detail elsewhere (9). Audio recordings were transcribed verbatim into Microsoft Word (Microsoft Corporation, Redmond, Washington), and transcripts were coded using ATLAS.ti version 5.0 (ATLAS.ti Scientific Software Development, Berlin, Germany), a qualitative data management software program. Coded data were reviewed for accuracy and examined for links to other codes. This “axial coding” process (10) connected code categories and identified relationships that could reasonably be taken to represent common themes. We used a constant comparison method (11) to allow for the discovery of similarities and differences in the data.

## Results

Physicians were mostly male (Table) and had actively engaged in patient care for an average of 16.7 years; a sizable percentage of their practices (37%) were made up of older adults. APPs were mostly female and had practiced an average of 26.2 years. Almost half (46%) of their practices consisted of older adults.

**Table:** Characteristics of Primary Care Providers and Their Practices, Focus Groups in Colorado, North Carolina, and Texas, 2007-2008

Characteristic	Physician (N = 28)^a^	APPs (N = 21)^a^
**Male sex**	78.6	19.0
**Age, y**		
≤44	53.6	9.5
45–64	39.3	90.5
≥65	7.1	0
**Number of years in practice, mean (SD)**	16.7 (12.8)	26.2 (8.5)
**Race/ethnicity**		
White	50.0	81.0
African American	0	9.5
Asian/Pacific Islander	25.0	0
Hispanic or Latina/Latino	17.9	4.8
Other	7.1	4.8
**Self-described specialty**		
Family medicine	57.1	71.4
Internal medicine	33.3	0
Geriatrics	0	23.8
Emergency medicine	3.7	4.8
Public health	3.7	0
**Specialty training in past 5 years**		
Geriatrics	10.7	35.0
Neurology	7.1	24.5
**Practice setting**		
Mostly urban	64.3	33.3
Mostly rural	35.7	66.7
**Dementia diagnosis usually made by . . .**		
Self	71.4	40.0
Specialist (from referral)	28.6	60.0
**Patients aged ≥65, mean % (SD)**	36.8 (23.1)	46.4 (29.8)
**Percentage of patients aged ≥65 with . . .**		
Alzheimer’s disease or related diagnosis	7.7	22.6
No notable memory impairment but who express concerns about brain or cognitive health	19.4	23.5

Abbreviation: APPs, advanced practice providers.

^a^ All values expressed as percentages unless otherwise indicated.

In response to the case study, providers emphasized physical health risks and most frequently recommended a healthful diet, physical activity, and medication adherence to achieve weight loss and diabetes control. They also recommended other preventive care services, illness surveillance, and exploration of the patient’s ability to follow recommendations.

Providers said they generally try to give patients a rationale for their recommendations, which most commonly focused on reducing risk for cardiovascular events and chronic disease management, but that time constraints limit their ability to do so. Despite the case study description of the patient’s mother having cognitive impairment and his own increased risk for cognitive impairment given his poorly controlled diabetes, few providers talked about risk of cognitive impairment; when they did, it was in the context of the benefits of physical activity on vascular risk, cancer prevention strategies, or screening for risky behaviors such as excess alcohol consumption:

Vigorous daily exercise . . . because it improves, basically, all the vascular risks which people in this age group face, especially someone with diabetes. It improves risk with dementia, which I’m sure you’d be concerned about, and actually reduces cancer risk as well. (Physician, focus group M)

I think I probably start just reviewing all this complication of diabetes. It sounds like you know about his mother’s health, so you can kind of go on that. I don’t mean to scare people, but sometimes when they have somebody they can look at and they have some complications that are similar to what they can have, it tends to sink in more. (APP, focus group J)

Providers were asked in consecutive questions about what advice they could give patients and what advice they actually do give patients. They tended to include the advice they actually give in response to both questions; for that reason, it was difficult to distinguish possible from actual advice, so responses to both questions were considered together for this analysis. Providers cited a range of recommendations to maintain cognitive health and avoid cognitive decline, including staying busy, volunteering, being socially engaged, being physically active, eating healthfully, doing puzzles or other games, reading, and learning new things or trying new activities. Providers also stressed disease management including controlling cholesterol, hypertension, diabetes, and other medical conditions.

If the time is available, encouraging social interaction, being out with people and discussing topics, encouraging mental stimulation as much as possible and reading, things like crosswords and puzzles and things like that. You know, trying to get people from just sitting at home alone watching [television]. (Physician, focus group I)

If they smoke, that’s a risk factor. If they’re alcoholic, that is a tremendous risk factor. . . . If they start looking at these things early on in their late 30s/early 40s, then they can start doing some things to help that from progressing. (APP, focus group A)

From a cardiopulmonary standpoint, no smoking, improve cardiorespiratory health, improve diabetes, self-management to lessen the end organ damage that can result from hyperglycemia. Maintain a healthier state so that an increased level of activity can continue into later life. (Physician, interview G)

Some providers felt Alzheimer’s disease is not preventable:

Stuff like Alzheimer’s, we can’t do anything about. Either you get it or you don’t. You can’t prevent it. You can’t slow it down, but the other risk factors for dementia, multiple strokes . . . related directly to blood pressure, diabetes, and obesity, you know, high cholesterol. Those we can control; stuff we can’t, we can’t. (Physician, focus group K).

Others cited evidence for the possibility of preventing cognitive decline or felt there was no risk in making recommendations based on weak evidence:

You know, the folks who have loved ones that maybe have dementia and they’ll ask “Is there some way I can prevent the disease from happening to me?” And I must admit, I think the data out there [are] inconclusive right now about doing those things, but there’s no harm in doing them. (Physician, focus group D)

Many PCPs said they do not routinely raise the issue of cognition with patients unless prompted, although a few did take a proactive approach to cognition such as using brief cognitive assessments to establish a baseline from which comparisons can be made over time:

For health maintenance, it’s not on my checklist that I talk to them about brain health, you know. (Physician, focus group D)

I have never initiated it [conversation about cognitive health or impairment] when the patient did not ask or when there was obvious family dementia or something that would bring it in closer. It’s not something I normally initiate in part because I don’t feel that we have that much to offer for it, aside from what I’m trying to do with their health in general. (Physician, interview B)

Let’s just speculate: less than 20% think about the mind and talk about preventive things to keep the mind to remain healthy. (APP, focus group L)

I think after a certain age, when they come in the first time, you include in your basic work either a mini-mental status exam or clocks so that, you know, baseline, where they are. So down the line, you can retest them periodically to see if either one of those is getting skewed. (APP, focus group C)

When PCPs were prompted to discuss what cues them to talk about cognitive health with patients, they most commonly talked about questions or concerns from patients or family members, family history of impairment, risk factors for impairment, or apparent impairment during the office visit.

The patients themselves report memory loss, or they can’t remember anything, or they are losing things. (Physician, focus group M)

Sometimes they will, in speaking to you, their verbal skills have changed. So I may then have them draw a clock or do a mini-mental status. I’ll have them keep a journal for the next 3 months and bring that journal to me and it’s amazing sometimes just in 3 months . . . the difference you can see in how they write. (APP, focus group A)

Time constraints were almost universally acknowledged as a barrier to discussions about cognitive health. Short office visits mean that only the most urgent concerns can be addressed. Discontinuity of care across health care settings and providers was seen as a barrier to developing relationships needed to discuss sensitive topics like cognition and to assess change over time:

The age-old thing, I’ve [got] 10 minutes, 15 minutes, 20 minutes, and you’ve got to do 5 or 6 things and this is probably not at the top of your list usually. (APP, focus group L)

A lot of my patients, I don’t have a trust established. I’ve never seen them before and I’ll never see them again. (APP focus group F)

Providers also described variability in patient attitudes and responses to recommendations about cognitive health as a barrier to discussing cognitive health during office visits:

I find compliance with my recommendations is directly proportional to the patient’s perceived fatality of the consequences present. (Physician, focus group M)

I think the recommendations [for preventing decline in cognitive functioning] are so generic that they’re kind of underwhelmed with the information. (Physician, focus group D)

## Discussion

PCPs reported addressing cognitive health indirectly as part of their usual clinical practice. Although they recognized the potential of a healthful diet, regular physical activity, and effective disease management to affect cognitive and physical health, they reported rarely mentioning cognitive health to patients unless prompted by the patients, their families, or the PCPs’ own observations of symptoms or risk for cognitive impairment. Providers’ self-reported practices are noteworthy, given the importance older adults place on maintaining cognitive health (2,3). Barriers to discussing cognitive health included system-level issues (eg, lack of time, not having a relationship with a patient) and patient-level issues (eg, adherence to recommendations for behavior change).

Our findings enhance those reported by Day et al (5), which indicated that less than half of respondents (40%) discussed cognition with adult patients who were not displaying cognitive impairment “often” or “very often.” Providers in our qualitative study offered recommendations for cognitive health that were similar to those reported by Day et al (5). Whether or not providers feel that evidence for these recommendations is sufficient, they may offer the recommendations when prompted because of other health benefits or their belief that following the recommendations would do no harm. Providers’ perception that evidence is insufficient to conclude that recommendations for specific activities will positively affect cognition are consistent with the National Institutes of Health State-of-the-Science Conference on Preventing Alzheimer’s Disease and Cognitive Decline, which produced a statement that insufficient evidence exists to conclude that modification of any risk factors will prevent Alzheimer’s disease or other causes of cognitive decline (12,13).

Our study has limitations. The generalizability of these findings is limited by the nature of the study sample, a convenience sample of self-selected participants from 3 states. Qualitative work in other geographic settings may indicate regional variations in perceptions and practices related to cognitive health in primary care. This study was conducted before the passage of the Patient Protection and Affordable Care Act (PPACA) (14). The PPACA initiated coverage for an annual wellness visit (AWV) that includes assessment of cognitive impairment by direct observation and consideration of patient and relevant others’ reports (§ 410.15). Provider practices may have changed since the introduction of AWVs. However, fewer than 2.3 million beneficiaries had an AWV in 2011 (15). Studies of the effect of AWVs on perceptions and practices will be useful for understanding how this change in policy has affected care related to cognitive health.

PCPs are stakeholders in efforts to address cognitive health from a public health perspective. This study adds to the literature by describing PCPs’ perceptions and practices and highlighting the challenges providers face when addressing older adults’ concerns about cognitive health. National efforts to address cognitive health will require partnerships between public health practitioners and PCPs. Communicating new evidence on cognitive health and engaging older adults in making lasting lifestyle changes recommended by PCPs and others may be practical ways in which public health practitioners can partner with PCPs to address cognitive health in health care settings.
